# Prevalence of parenthood among hospitalized adult patients with severe mental illness: a quantitative data analysis

**DOI:** 10.3389/fpsyt.2024.1386842

**Published:** 2024-07-15

**Authors:** Anna Havelková, David Havelka, Kateřina Koros Bartošová

**Affiliations:** ^1^ Department of Psychology, Faculty of Arts, Masaryk University, Brno, Czechia; ^2^ Children’s Psychiatric Hospital Opařany, Opařany, Czechia; ^3^ Department of Psychology, Faculty of Education, Masaryk University, Brno, Czechia

**Keywords:** offspring of patients, COPMI, prevalence, parenting, SMI, medical records

## Abstract

**Introduction:**

In the Western world, more than one-third of the patients of productive age hospitalized for severe mental illness (SMI) are parents. Each of their offspring is exposed to several stressors related to their parent’s illness and hospitalization, which puts them at an increased risk of developing mental health problems. In the Czech Republic, no statistics are currently available about the families of patients with SMI, inpatients who are parents, or data about their children (ages ≤18 years). Therefore, our research aim was to describe the prevalence of parenthood among hospitalized patients with SMI, assess the number of children and determine the extent to which offspring information was present in medical records.

**Methods:**

Quantitative data from medical records (2,768 patients, aged 18–63 years, hospitalized for SMI between 2017 and 2020) from two large inpatient psychiatric facilities were examined. Parental information, demographic characteristics, number of children, and other available data were collected.

**Results:**

The prevalence of parenthood among inpatients with SMI was 34.6%. Parenthood was most prevalent among female patients and patients with recurrent depressive and bipolar disorders. The total number of offspring in 957 patient-parents was 1781 (41.7% minors under the age of 18). Information on parenthood was available in 99.7% of cases; information on the age of offspring, custody, and sociodemographic situation varies, being included in 73% to 89.7% of the medical records (some details were more frequently recorded than others).

**Discussion:**

The data obtained may help to better understand and address the specifics of these families and thus serve as a basis for the development of prevention programs.

## 1 Introduction

Parents comprise a significant proportion of patients with severe mental illness (SMI) (1). In this paper, consistently with other studies (2), the term SMI includes these diagnoses: schizophrenia spectrum disorders, recurrent depressive disorder and bipolar affective disorder. Children of parents with mental illness are at high risk for also developing a mental disorder, yet they receive little attention (2). Meta-analyses show that children of parents with SMI have a high risk of transgenerational transmission of psychopathology, with up to a 50% risk of developing mental illness and a 32% risk of developing severe mental illness (3). These risks are due to not only the hereditary burden (a specific risk factor) but also several non-specific risk factors (poor socioeconomic family situations, relatively high parental unemployment rate, parental stress, stigmatization of families with mentally ill members, more frequent placement in foster care, etc.) (4).

Regarding the mechanisms of risk in children, it appears that risk is determined by the severity and chronicity of psychopathology and differences in parental personality, genetic characteristics, coping style, and social circumstances, rather than the parent’s diagnosis itself (5, 6). During parental hospitalization, children may have difficulty in coping with their circumstances, separation from their parent, the parent’s condition, and their painful and negative emotions, including grief (7).

In terms of sociodemographic characteristics, compared to other children, those in families in which a parent has a diagnosed mental illness are two times more likely than others to live in a lower income family household (e.g., 8, 9), be unemployed (8, 10) and have lower education levels (8, 11). Children of parents with mental health disorders are also more likely than those in the general population to live with only one parent (10, 11). For example, only 10% (5 of 50) of the mothers in the study by Benders-Hadi et al. (12) were married. In order to provide appropriate supportive interventions, we need to look at the socio-demographic circumstances as well.

Prevention programs for the children of parents with mental illness have been implemented in some other countries (Australia, Norway, Finland, Denmark, The Netherlands, USA, etc.), and the related research shows that these programs reduce children’s risk of developing mental illnesses (13, 14), sometimes by up to 40% (3). Other studies have demonstrated that preventative intervention programs protect both parents with SMI and their offspring (15). However, appropriately targeting support for families with a parent with SMI requires knowing the prevalence of parenting among patients and having access to detailed information about children living in families with a parent with mental illness and the factors that influence the mental health of all family members. In the Czech Republic, no demographic statistics are currently available about the families of patients with SMI and inpatients who are parents.

We decided to use a quantitative study of medical records to investigate parental rate among hospitalized patients with SMI as used in similar studies of clinical populations (e.g., 12). However, there are several ways to examine the prevalence of parenting in the literature. For an overview of different methodological approaches and reported prevalence rates, see our recent review (16). Studies of clinical populations can take the form of census studies in which either professionals collect data by completing questionnaires about patients (17, 18) or the patients complete questionnaires about themselves and their children (19, 20). In studies similar to the present one, the reported prevalence of parenthood ranged from 25–36% among inpatients with mental illness (12, 17, 20) and up to approximately 50% among outpatients (21, 22).

Therefore, the aims of this study were a) to determine the prevalence rate of parenthood among SMI patients and to obtain detailed information about their offspring and b) to determine the extent to which family characteristics are included in the medical records of patients with SMI, and c) identify relevant family demographics and characteristics data that should be part of psychiatric patients medical files to design more effective and individualized interventions. Therefore, we formulated the following research questions:

- What was the prevalence of parenthood in patients hospitalized for SMI?
- What were the sociodemographic characteristics of the families of these patients?
- Was information on the age and mental health of the children of parents with SMI included in the medical records?
- How many parents with SMI had children in their custodial care?

## 2 Methods

We defined SMI for research purposes based on the ICD-10 diagnoses, that is, schizophrenia spectrum diagnoses (F2), recurrent depressive disorder (F33), and bipolar affective disorder (F31).

### 2.1 Design

This cross-sectional study was conducted using data related to inpatients in two collaborating general psychiatric hospitals with 500- and 1,000-bed capacities in districts and towns in the Czech Republic. Data were obtained by analyzing the medical records of patients with SMI hospitalized between 2017 and 2020.

### 2.2 Procedure

Two collaborating psychiatric inpatient facilities were contacted for data collection. Both facilities are urban psychiatric hospitals with acute care and rehabilitation programs and a capacity of approximately 500 to 1000 patients. Data were collected in March 2022 by one authorized employee at each facility (two individuals in total). From an anonymized patient list, the authorized staff member identified all patients who, at the time of hospitalization, were between the ages of 18 and 65 years and had been admitted with SMI diagnoses at any time between January 1, 2017, and December 31, 2020. In cases of patients who had repeated hospital admissions within those years, we only included the data that corresponded to one of those admissions. In this case, the number of admissions was recorded and data from the last hospitalization were obtained. From this pool of patients, those with indications of parentage in their medical records were selected. For these parent patients, additional data were obtained from medical records, specifically most often from the part of social and family histories documented by psychiatrists at admission.

### 2.3 Ethics

The data were collected after obtaining approval from the ethics committees of Masaryk University and the cooperating medical facilities. Data collection and anonymization were performed by an authorized employee at a medical facility. Since only anonymous statistical data were provided in the study, informed consent from individual patients was not required according to Czech legislation. The researchers worked only on statistical data and could not identify individual patients.

### 2.4 Data collection

Data were obtained from the electronic medical records system, specifically from medical examinations and psychosocial histories documented by psychiatrists at admission. The data consisted of the sex, age, main diagnosis, and parental status of all patients with relevant diagnoses who were hospitalized over a 3-year-period. For patients who were parents, additional data were subsequently collected from the medical records: the number and length of the last hospitalization, suicide attempts, marital status, family situation, education, disability pension, number of children and their ages, children’s health (psychological/psychiatric problems only), their relationship to people with whom they lived, and who took care of the minor children during hospitalization.

### 2.5 Data analyses

Quantitative data were analyzed using SPSS 27 software with descriptive statistics, and the chi-square test was used to detect differences in the prevalence of parenthood between the groups by sex and diagnosis. Information on the offspring (health status, relationship with parents, and information about caregivers) varied in the medical records. Content analysis of that information was conducted by two independent researchers (A.H. and D.H.). The occurrence of certain words was quantified into categorical variables according to established coding rules (e.g., the item ‘caregivers’ was coded into categories of mother/father/grandparents/institutional care, etc.). Inter-rater agreement was tested on a selected sample. As a result of good parameters (Cohen Kappa varied from 0.91 to 0.96)^1^ and the nature of the data, which showed clear and unambiguous categories, inter-rater reliability was not determined for the full sample (due to sample size and staffing). The resulting univariate categorical variables were used in chi-square analyses to determine the differences between the parents’ main diagnosis and the presence of a child in the household.

## 3 Results

### 3.1 Sample

The sample consisted of data on 2768 adult inpatients (57% men, 43% women) aged between 18 and 63 (M = 42.12; SD = 11.48) at the time of their last hospitalization. Patients had a main diagnosis of schizophrenia (F20) in 42%, other psychotic disorders (F2x) in 38%, recurrent depressive disorder (F33) in 11%, and bipolar affective disorder (F31) in 8% of cases.

### 3.2 Prevalence of parenthood

Of the 2,768 patients, 957 were parents (34.6%); 63.1% of whom were women and 36.9% were men. Information on parenthood was missing in 9 cases (0.3%). In the group of patient-parents, 46.2% of them had a minor child under the age of 18 at the time of hospitalization. The parents of minor children (*n* = 446) had 666 minor children.

The prevalence of parenthood was higher among hospitalized women (50.8%) than among men (22.5%) (x2 = 239.44; df = 1; *p*< 0.001). Significant differences between patients with and without children were also observed across diagnoses. Patients with depressive and bipolar disorders were more likely to be parents than patients diagnosed with schizophrenia. Further details are presented in **Table 1**.

**Table 1:** Differences between patients-parents and patients without children by sex and main diagnosis.

	Patient-parents *n* (%)	Childless patients *n* (%)	Total (*n)*	Statistical analyses
Sex				
Men	353 (22.5%)	1217 (77.5%)	1570	x2 = 239.44
Women	604 (50.8%)	585 (49.2%)	1198	df = 1
				*p* < 0.001
				Phí = -0.295
Main diagnosis				
Schizophrenia (F20)	243 (20.7%)	932 (79.3%)	1175	
Other psychotic illness (F2x)	422 (39.9%)	636 (60.1%)	1058	
Depressive disorder (F33)	181 (59.3%)	124 (40.7%)	305	x2 = 219.78
Bipolar disorder (F31)	111 (50.2%)	110 (49.8%)	221	df = 3
				*p* < 0.001
				Cramer V = 0.282

Diagnosis according to ICD-10, F2x=other psychotic disorders from category F2, except schizophrenia.

### 3.3 Sociodemographic characteristics of patient parents

Nearly half of the hospitalized parents had at least a secondary education level of high school completion (they earned a diploma) or higher. Most parents were either employed (44.0%) or received disability pension benefits due to psychiatric indications (43.3%), and 6.6% of parents were unemployed. Almost half (47.9%) of the hospitalized parents lived in a partnership or marriage, a quarter (25.4%) lived alone, while the remainder lived with other relatives (most often their parents, siblings, or adult children) or, less often, in shelters, hostels, and nursing homes (6.7%) or homeless (2.6%). Legal capacity (ability to take independent legal action) had been restricted for 8.1% of patients who were parents. Information on education was present in the medical records of 81% of the patient-parent cases, on employment in 73%, on legal capacity in 82%, and information on housing in 81% of cases. See **Table 2** for full details of the percentages of available information.

**Table 2:** Sociodemographic characteristics of patient-parents (*n* = 957).

	Frequency *(n)*	Percentage *(%)*
Highest level of education		
Learning disability elementary school	20	2.6
Elementary school	96	12.4
Vocational school	291	37.5
High school	262	33.8
University	106	13.7
Missing	182	
Sources of income		
Employed	306	44.0
Unemployed	46	6.6
Disability pension due to psychiatric indication	301	43.3
Disability pension due to somatic indication	29	4.2
Parental leave	10	1.4
Retirement	3	0.4
Missing	262	
Legal capacity		
Legally capable	719	91.9
Restricted legal capacity	63	8.1
Missing	175	
Housing arrangements		
Alone	197	25.4
With partner/in marriage	372	47.9
With other relatives	136	17.5
Supported housing	52	6.7
Homeless	20	2.6
Missing	180	

### 3.4 Offspring of hospitalized SMI patients

#### 3.4.1 Age of inpatients’ offspring

The hospitalized patients had a combined total of 1,781 offspring. The mean number of children in the family was 1.9 (*SD* = 0.86) and ranged from 1 to 6. The age (or, at least, information about adulthood) of the offspring was reported in 89.7% of cases. Almost 60% of the offspring were adults and one-third were school-aged. **Table 3** provides the detailed information.

**Table 3:** Age of offspring of hospitalized patients.

Age of offspring	*n*	Valid percentage (%)	Cumulative percentage (%)
0–2 years	70	4.4	4.4
3–5 years	115	7.2	11.6
6–14 years	368	23.1	34.7
15–17 years	113	7.1	41.7
Adult	930	58.3	100.0
Missing	185		
**Total**	1781		

#### 3.4.2 Mental health of inpatients’ offspring

Explicit information on the mental health of the inpatients’ offspring (“healthy” or a specific illness) was given in only 16.4% of cases. The frequency of individual mental disorders is presented in **Table 4**; somatic diagnoses were omitted due to the scope of the article. Seven offspring were deceased (three had died by suicide).

**Table 4:** Frequency table of mental illness in offspring (*N*=1781).

Information on the offspring’s health	*n = 292*
**Healthy**	212
**Mental disorders**	
Psychotic disorders	19
ADHD	11
Neurotic, stress-related and somatoform disorders	9
Mood disorders	8
Addiction	8
Mental retardation	8
Autism spectrum disorders	5
Unspecified mental difficulties	5
**Death**	7

### 3.5 Children of hospitalized patients-parents

#### 3.5.1 Care for children of hospitalized parents

Of the 1,596 offspring for whom we knew their age, there were 666 minors under the age of 18. The mean age was 9.1 years (*SD* = 4.84), ranging from 0 to 17 years. Half of these children were under the care of both parents. Forty-two children (6.2%) lived with their hospitalized parents alone (without the presence of the other parent, spouse, or other relatives). All single, hospitalized parents were mothers. We know from medical records that in the case of two mothers, their child was placed in transitional foster care for the duration of their hospitalization. However, no information was available about the other 40 children. Children who were not living with any parent (i.e., living with another relative, in foster care, or with adoptive parents) accounted for 9.2% of our sample. For 13.7% of the children, we had no data. **Table 5** provides additional information on this topic.

**Table 5:** Care of minor children (n = 666) of hospitalized parents.

Where minor children live	*n*	Percentages (%)	The patient is the mother (n)	The patient is the father (n)
In a family with both parents	336	50.5	226	110
With a hospitalized single parent	42	6.3	42	0
In the care of a hospitalized parent living with other relatives	32	4.8	31	1
In shared parenting	13	2.0	9	4
In the care of a second parent	87	13.1	45	42
In the care of other relatives	26	3.9	23	3
In foster care/adoption	31	4.7	29	2
In institutional care	4	0.6	4	0
No information on where the child lives (not in the care of a hospitalized parent)	91	13.7	19	72
Died	4	0.6	4	0

#### 3.5.2 Living in a shared household: differences between sex and parental diagnosis

We divided the sample of children into those living with a hospitalized parent, which included children living with both parents, with a hospitalized parent only, and in shared parenting (63.6% of minor children), and those not living with a hospitalized parent (36.4%). If a child lived with a hospitalized parent, the child was significantly more likely to have a hospitalized mother (72.8% of cases) than a hospitalized father (27.2% of cases). If the mother was hospitalized, she was 71.3% likely to live with her child, whereas the likelihood for the father was only 49.1%. These differences are statistically significant (x2 = 32.14; df = 1; *p* < 0.001; Phi = -0.220).

Children of parents hospitalized with schizophrenia were cohabiting with their parents in 47.7% of cases, children of parents with bipolar disorder in 53.8% of cases, and children of parents with other psychotic disorders (72.0%) and recurrent depressive disorder (74.5%) were even more likely to live together with the hospitalized parent. The differences in the parent’s main diagnosis and the presence of the child in the household were statistically significant (x2 = 38.99; df = 3; *p* < 0.001; Cramer V = 0.239).

#### 3.5.3 Number and duration of parental hospitalizations

The parents of children were hospitalized at an average of 1.7 times (*SD* = 1.58, mode = 1) during the 3-year follow-up period, and the number of hospitalizations varied from 1 to 16. The mean length of the most recent hospitalization was 61.8 days (*SD* = 66.79), ranging from 1 to 901 days.

#### 3.5.4 Suicidality of hospitalized parents of minor children

Among the hospitalized parents with children, the data indicates that there was an 8% prevalence of suicide-attempt history, with 2.4% of the patients having a history of repeated suicide attempts.

#### 3.5.5 Relationship between hospitalized parent and child

Information about the relationship between the hospitalized parent and child was purposely searched in medical records. Some references to a relationship were provided in 39.5% of the 666 cases. These statements were grouped into 4 categories using content analysis: “good relationship” (195 times), “occasional contact” (22 times), “not in contact” (43 times) and “conflict relationship” (3 times).

## 4 Discussion

The prevalence of parenthood among hospitalized patients with SMI in our study was 34.6%. This result is similar to the 38.5% prevalence rate reported in a previous US study (12). That study’s patient participant sample also included minor and adult offspring; however, all of the patients were women, and women generally tend to have a higher parenthood prevalence. For example, in Östman and Hansson’s (20) study, 75% of parents with SMI were women, and an Australian national study (8) found 56.2% of the patients with children were women, and only 25.9% of the men were parents. The prevalence of parenthood in our study was also higher among hospitalized women (50.8%) than among men (22.5%).

In comparison to other methodologically similar studies (19, 20), the prevalence identified in our sample was slightly higher (28% vs. 34.6%). This difference may be explained by the moderately different distribution of hospitalized patients; a significant proportion of parents in our study were hospitalized for depressive disorder, whereas in other studies, a larger proportion of the sample consisted of patients with schizophrenia, who were parents less often (again confirmed by our results). However, it cannot be said that there are no parents among patients with psychotic disorders or that they do not live in the same household as their children. Half of the minor children whose parents were hospitalized for schizophrenia lived in the same household as the parent. The percentage of parents with other psychotic disorders co-habiting with their children was 72.0%. This is an even higher percentage than that reported by Howe et al. (17) (34.0% to 56.4% of minors living with a parent with a psychotic disorder from 2008 to 2011).

In addition to increased psychological demands, the vulnerability of the offspring of parents who are inpatients is magnified by a worsening of the family’s socioeconomic status. SMI parents are more likely to be unemployed, and SMI is a risk factor for living below the poverty line (9). The patients in our sample had lower education levels (52.5% of parents had lower education than secondary with high school diplomas versus 44.1% in the general population (23) and their unemployment rate was twice that of the general population (6.6% vs. 2–3%, see 24). In addition, 47.5% of the parents received a disability pension (43.3% because of psychiatric indications) meaning that their ability to work was limited or completely inhibited in almost every second case. This finding illustrates the significant effect of SMI on family life. Support aimed at parental employability could potentially lead to a more stable family situation and other family benefits.

In our study sample, the majority of the offspring were over 18 years old, which may be explained by the wide age range of the patient sample, because we aimed to cover the widest possible spectrum of potential parents of minor children. The analyses also revealed that medical records of 99.7% of SMI patients had at least basic information about parental status in their records, and in a significant proportion of cases, additional information about their offspring (e.g., ages or information about the adulthood of offspring in 89.7%). These data are in contrast with the UK study conducted by Gatsou et al. (25) in which parental status was presented in 62% of the cases only. Our data indicate a well-established and routinized medical assessment of parenthood during the admission process to collaborating facilities.

From the perspective of mental health problem prevention, data on the mental health of the offspring were only sporadic in the medical records (only 16.4% of the offspring). In Manderson and McCune’s (26) study, an inquiry into children’s welfare was carried out in 24.2% of cases. These data may indicate an insufficient focus on investigating the mental health of offspring, which is an important first preventative step in identifying families in need of additional support. However, it is important to consider that patients are often admitted in the acute phase of the illness when it is not possible to collect an adequate amount of objective information.

Minors in our study shared a household with the hospitalized parent in 63.6% of cases, which is a smaller percentage compared to a recent Norwegian study (27), in which 76.2% of minor children cohabitated with a parent with mental illness. This finding can be explained by two factors: Norwegian researchers more frequently included parents with affective disorders than we did in this study, and the topic has received more focus for a longer period in Norway. There are established intervention programs and dedicated legislation—The Norwegian Health Personnel Act of 2010—postulating, among other things, a legal obligation for health professionals to investigate parental status and offer appropriate support to offspring when needed.

Children were more likely to live with hospitalized mothers than with fathers. This finding may be related to mothers perceiving parenthood as a part of their identity (12, 28, 29). This is another reason why we should address the topic of motherhood in the treatment of hospitalized women, because it can serve as a protective factor against risky behavior (e.g., suicidality and remaining in harmful relationships) (29, 30). The impact of fatherhood on male patients’ identity is a less researched topic, but it appears that men also perceive fatherhood as part of their identity, and they feel proud of and concerned about their offspring (31–34).

Hospitalization also affects other parents who take over most of the childcare responsibilities. Half of the children of the patients in our sample lived in a family with both parents (only 38.9% in the Norwegian study, in which more children were raised through shared parenting - see 27). Forty-two minors (6.3%) lived in a family with a hospitalized single parent (mother) without any other adults. Information on who cared for the children during the mother’s hospitalization was available for only two children (a facility for children in need of immediate support). Within this framework, there is room for increased inquiry into the family situation of hospitalized parents without an extended family background, and collaboration between clinicians and other organizations is essential. Children of single parents can be negatively affected not only by the absence of a parent but also by the difficult situation they are in during the hospitalization of their parents. Moreover, mothers may postpone their hospitalization out of concern for their child’s safety, as indicated by Diaz-Caneja and Johnson (35).

We also aimed to map the frequency of risk factors for the development of mental health problems in children whose parents are hospitalized. Risk factors for transgenerational transmission of psychopathology include younger child age (5). In our sample, 12.9% of children were in the “under-5 years old” category. Additionally, the parent’s diagnosis per se may not play such a large role; the severity of the illness (suicidality and psychotic symptoms) and the frequency and chronicity of the illness are more impactful (36). The average parent of a minor child in our sample was hospitalized approximately twice over three years for approximately two months on each occasion, which is almost an entire school term when the child is without one parent and the other parent or relative must take over care. Suicide attempts occurred in 8% of cases in our sample in the history of parents of minor children, but this information may be misleading because it is not clear whether the attempt occurred before or after the birth of the child. However, this is an important factor, because the likelihood of repeated suicidal behavior increases with a history of suicide attempts (37), placing children at an increased risk of developing mental health problems. On the other hand, according to research, being a parent can be a protective factor against suicide (38); therefore, addressing the topic of being a parent and parenting in clinical work may have great protective benefits not only for hospitalized parents but also for their offspring.

Research has shown that a relatively large percentage of families with parents with SMI present a range of risk factors for the development of mental health problems in children (2, 3, 9). Increased attention and support should be given to these families, because preventing the development of mental disorders in the next generation is an important part of the agenda of mental health professionals. If we, as professionals, realize that the patient is part of a family system that is influenced by him/her and reciprocally influences him/her, we can dramatically improve both the patient’s treatment and prevent the development of mental health problems in his/her offspring.

### 4.1 Study strengths and limitations

In our research, we attempted to address a question that has received only minimal attention in the Czech Republic thus far and to provide up-to-date data mapping of the current situation. Prevalence research is the first step, preceding further studies aimed at mapping the needs of the families of patients with SMI. Based on this information, it is possible to integrate parenthood and parenting into the clinical assessment, treatment, and psychosocial rehabilitation of patients and their families. Similar to other countries, this study can be used as a basis for the implementation of prevention programs.

However, this study had several limitations. The first is a certain degree of simplification in the definition of SMI to diagnostic categories only because the design of the data collection did not allow for a more detailed specification of SMI, for example, according to functional criteria (Global assessment of functioning score – GAF, presence of psychosis, and duration of illness). Since medical records do not use a system of functional criteria, diagnoses are central. Data collection was conducted in two similar types of inpatient psychiatric facilities; thus, we cannot comment on the prevalence of parents among patients with SMI throughout the Czech Republic. Future studies would be enriched by information from outpatient and rural forms of care. The current data were only cross-sectional; therefore, it is not possible to generalize the results over time or, for example, to look for causal links between parental characteristics and their impact on the child. The collected data did not allow us to compare the sociodemographic characteristics of parents and non-parents.

The data were also from admission assessments in medical records only and, thus, might be influenced, for example, by the admission of a patient in an acute state of illness or by the recording patterns of individual clinicians. It would be interesting to complement future research with subjective views and attitudes of patients with SMI. Future (qualitative) research could reach out directly to patients for data collection and to explore their experiences and support needs, so that prevention and support programs could be tailored better for patients.

### 4.2 Recommendations for practice

This study provides several recommendations for clinical practice. The first general recommendation is a greater emphasis on parenthood and parenting in the medical assessment and treatment of patients with SMI, because it may increase motivation and compliance during the recovery process. During family history mapping, clinicans should explore in detail the relationship between the patient and their child and the care of children of hospitalized patients and offer support services to children as needed. Similarly, the mapping of the health status of offspring can serve as a safety net and, with appropriate supportive interventions, prevent the development of mental health problems in the next generation. In addition, stronger collaboration and liaison between adult and child psychiatry and clinical psychology practices can facilitate and speed up cooperation and prevent the development of serious problems in children.

Studies and materials evaluating the impact and benefits of working with families and parents should be incorporated into the curriculum of professional mental health education. Inspired by Norway (The Norwegian Health Personnel Act of 2010), we recommend consideration of building legislative endorsement of supportive interventions to be offered to families and family members who are at risk for developing mental health problems.

## 5 Conclusion

More than one-third of patients hospitalized for SMI were parents. These patients’ children (often minors) are exposed to several psychosocial stressors associated with their parents’ illnesses and hospitalization, in addition to the hereditary burden. Parental status is well registered in medical records, unlike other more detailed information, such as the child care during a parent’s hospitalization. This study aimed to highlight this topic and provide up-to-date data on the prevalence of parenthood in patients with SMI. This study may provide a background for further enhancing clinical assessments and implementing preventative interventions in families with risk factors. As more details about patients’ family characteristics—and their individual roles in their families and communities—become available, rehabilitation and recovery will become both more inclusive and more personalized.

## Data availability statement

The datasets for this article are not publicly available due to concerns regarding patient anonymity. Requests to access the datasets should be directed to the corresponding author, zachova.anna@gmail.com.

## Ethics statement

The studies involving humans were approved by ethics committee of Masaryk University. The studies were conducted in accordance with the local legislation and institutional requirements. Written informed consent for participation was not required from the participants or the participants’ legal guardians/next of kin in accordance with the national legislation and institutional requirements.

## Author contributions

AH: Conceptualization, Formal analysis, Methodology, Resources, Visualization, Writing – original draft, Writing – review & editing. DH: Conceptualization, Data curation, Funding acquisition, Writing – original draft, Writing – review & editing. KK: Project administration, Software, Supervision, Validation, Writing – review & editing.

## Footnotes

1 Variable *Caregivers*: kappa = 0.91, SE = 0.058, t = 10.39, p < 0.001; variable *Relationship:* kappa = 0.95, SE = 0.052, t = 8.48, p < 0.001; variable *Health status:* kappa = 0.96, SE = 0.052, t = 8.48, p < 0.001.
